# *Wolbachia* infection and its effects on mitochondrial ND5 gene variation in *Aedes albopictus* from Shandong Province

**DOI:** 10.3389/fmicb.2026.1943745

**Published:** 2026-10-06

**Authors:** Tong Cai, Qiaoyi Liu, Ruoxi Xu, Zu Ma, Wenjie Liu, Yingnan Han, Qintong Sun, Yan Liu, Xinyue Cao, Taoyu Wu, Tongyan Zhao, Liping Yang, Wenkui Sun, Hengduan Zhang, Xuejun Wang

**Affiliations:** 1 Shandong Center for Disease Control and Prevention, Shandong Institute of Preventive Medicine, Shandong Provincial Key Laboratory of Intelligent Monitoring, Early Warning, and Prevention of Infectious Diseases, Jinan, China; 2 Department of Epidemiology, School of Public Health, Cheeloo College of Medicine, Shandong University, Jinan, China; 3 School of Public Health, Shandong First Medical University, Jinan, China; 4 State Key Laboratory of Pathogen and Biosecurity, Academy of Military Medical Sciences, Beijing, China

**Keywords:** *Aedes albopictus*, mtDNA-endosymbiont association, ND5, selective sweep, *Wolbachia*

## Abstract

**Introduction:**

*Wolbachia*, a matrilineally inherited endosymbiont, has the potential to influence host mitochondrial DNA (mtDNA) diversity through cytoplasmic hitchhiking and selective sweeps. *Aedes albopictus* naturally harbors *Wolbachia*, making it an ideal model for studying host-symbiont interactions. However, *Wolbachia* infection in Shandong Province and its effects on host mitochondrial ND5 gene remain poorly characterized.

**Methods:**

A total of 388 female *Ae. albopictus* were collected from 16 cities across 5 climatic zones in Shandong. *Wolbachia* infection was detected using *wsp*-based typing primers, and geographical distribution and climatic responses were analyzed. ND5 gene was amplified and sequenced. Haplotype networks, phylogenetic trees, and *Wolbachia*-ND5 association plots were constructed. Correlation analyses, neutrality tests, and AMOVA were performed to assess the associations between *Wolbachia* infection and ND5 genetic diversity.

**Results:**

The overall *Wolbachia* infection rate was 92.8%, with coinfection A&B as the dominant type (81.4%), followed by “*w*AlbB only” (6.2%) and “*w*AlbA only” (5.2%) infections. The infection rate varied notably among cities (61.5–100%) and climatic regions (77.6–98.7%). The overall infection and coinfection rates were negatively correlated with temperature, while the “*w*AlbA only” rate was positively correlated with precipitation. A total of 21 ND5 haplotypes, one *w*AlbA haplotype, and four *w*AlbB haplotypes were identified. Hap1 and Hap2, the ND5 haplotypes more closely associated to *Wolbachia*, were predominant and most broadly distributed among populations. Compared with the *Wolbachia* uninfected group, ND5 nucleotide diversity was reduced in the infected, “*w*AlbB only,” and coinfection groups, while haplotype diversity was reduced in the “*w*AlbA only” group. Neutrality tests showed significantly negative Tajima’s D and Fu’s Fs for the infected and coinfection groups, suggesting recent population expansion or selective sweeps.

**Discussion:**

This study revealed a high *Wolbachia* prevalence in Shandong *Ae. albopictus*, with coinfection A&B dominant and notable regional heterogeneity. The low genetic diversity of both *Wolbachia* and ND5, combined with reduced diversity in infected groups and significant negative neutrality test values, supported *Wolbachia*-associated selective sweeps on host mtDNA, although findings for the “*w*AlbA only” group should be interpreted cautiously due to low sample size. This study provided opportunities to better understand host-symbiont interactions, showing potential value for vector monitoring and control.

## 1 Introduction

*Wolbachia* is a Gram-negative endosymbiont widely distributed in arthropods. It was first identified in the reproductive tissues of *Culex pipiens pipiens* in 1924 (Hertig and Wolbach, 1924), and it was estimated that approximately 28% of mosquito species and 66% of insect species naturally carry *Wolbachia* (Zheng et al., 2019; Zhang et al., 2022; Zang et al., 2024). *Wolbachia* primarily colonizes reproductive tissues and can manipulate host reproduction through various strategies, including cytoplasmic incompatibility (Werren et al., 2008; Moretti and Calvitti, 2013), feminization (Rousset et al., 1992), parthenogenesis (Weeks and Breeuwer, 2001; Ma and Schwander, 2017), and male killing (Francis et al., 1998). Vertical transmission is the main route of *Wolbachia* spread within host populations, followed by horizontal transmission, and both modes collectively facilitate the rapid dissemination of *Wolbachia* through host populations (Werren et al., 1995).

Mitochondrial DNA (mtDNA) is a double-stranded covalently closed circular molecule that possesses high copy number, conserved nucleotide composition, rapid evolutionary rate, and strict matrilineal inheritance, which collectively make it a popular molecular marker for species identification, phylogenetic and phylogeographic analysis, and population genetics analysis (Ma et al., 2025). Cytochrome c oxidase subunit I (COI) and NADH dehydrogenase subunit 5 (ND5) are the two most commonly used mitochondrial markers in mosquitoes, among which ND5, owing to its faster evolutionary rate, provides higher resolution for detecting recent population events, such as divergence of closely related species, population expansion, and invasion (Kamgang et al., 2011; Kang et al., 2023). Notably, because *Wolbachia* and mtDNA are both matrilineally transmitted, the spread of *Wolbachia* within populations can elevate the frequencies of linked mtDNA haplotypes through a mechanism known as cytoplasmic hitchhiking, thereby decreasing mtDNA diversity (Kambhampati et al., 1992). At the intraspecific level, the rapid spread of *Wolbachia* may result in selective sweeps of the initially associated mtDNA variants (Charlat et al., 2009; Jackel et al., 2013). At the interspecific level, it may drive mitochondrial gene infiltration among populations of the same genus (Narita et al., 2006; Gompert et al., 2008; Jackel et al., 2013). These effects may introduce biases or even errors in mtDNA-based phylogenetic analyses (Hurst and Jiggins, 2005; Narita et al., 2006). Therefore, clarifying the association between *Wolbachia* infection and mtDNA variation is essential for the correct interpretation of host population genetic structure. However, to our knowledge, few studies have investigated the effects of *Wolbachia* on ND5 variation.

*Aedes albopictus*, one of the most invasive species, serves as a competent vector for a range of arboviruses, such as Dengue virus (DENV) (Guo et al., 2013; Guo et al., 2016), Zika virus (ZIKV) (Jian et al., 2022), Tahyna virus (TAHV) (Cai et al., 2023), and Chikungunya virus (CHIKV) (Vega-Rua et al., 2020; Veronesi et al., 2023), posing significant public health concerns. Wild *Ae. albopictus* naturally harbor two *Wolbachia* strains, *w*AlbA and *w*AlbB, with a high global natural infection rate of 87.1% (95% CI: 78.0–92.8%) (Gebremariam et al., 2024), making this species a good model for studying host-symbiont genomic interactions. Shandong Province, located in the eastern coastal region of China, encompasses diverse geographical units such as coastal areas, inland plains, hills, and mountains, resulting in marked spatial heterogeneity in climatic and ecological environments (Li et al., 2022; Meng et al., 2025). Specifically, eastern Shandong climatic region is strongly influenced by the ocean, characterized by a relatively mild, humid, and windy climate; northwestern Shandong climatic region exhibits a continental climate, characterized by high temperatures and semi-humid to semi-arid conditions; southwestern Shandong climatic region lies within a region of strong seasonal precipitation, characterized by high temperatures, abundant sunshine, and drought-flood cycles; central Shandong climatic region, dominated by mountains, hills, and valleys, shows notable local climatic variation and is generally cool and humid; south-central Shandong climatic region, with terrain dominated by low hills, is characterized by a mild, low-wind, and humid to semi-humid climate. Therefore, Shandong Province provides a diverse and representative setting for fine-scale studies on *Wolbachia* infection status, climatic responses, and host-symbiont genomic interactions. Yet, few such studies have been conducted here.

In this study, natural populations of *Ae. albopictus* covering all five climatic zones and all 16 cities of Shandong Province were used as study samples. The *Wolbachia* infection rate, infection types, geographical distribution characteristics, and climatic responses were first characterized. Subsequently, mitochondrial ND5 haplotypes, *Wolbachia* haplotypes, and the coevolutionary associations between them were analyzed. Finally, host mitochondrial ND5 genetic diversity under different *Wolbachia* infection backgrounds, as well as the *Wolbachia*-associated selective sweeps, were evaluated. This work enhanced our understanding of how symbiotic bacteria shape host mitochondrial genetic diversity and offered baseline data for region-specific vector surveillance and the design of *Wolbachia*-based biocontrol strategies in Shandong Province.

## 2 Materials and methods

### 2.1 Sample collection

Sixteen natural populations of *Ae. albopictus*, collected from 16 cities in Shandong Province during May–October 2024 (the period of high adult mosquito density) (Sun et al., 2026), were used in this study. The cities including Yantai (YT), Qingdao (QD), Weihai (WH), Zibo (ZB), Jinan (JNA), Taian (TA), Weifang (WF), Jining (JNI), Rizhao (RZ), Dongying (DY), Binzhou (BZ), Dezhou (DZ), Liaocheng (LC), Linyi (LY), Zaozhuang (ZZ), and Heze (HZ). Adult mosquitoes were collected using BG-traps or light traps. All specimens were morphologically identified as *Ae. albopictus*, and female adults were selected for subsequent analyses. A total of 388 specimens were included in this study and stored at −80 °C prior to experimentation. The specific information of the sampling sites and dates was provided in Supplementary Table S1.

### 2.2 Genomic DNA extraction

Genomic DNA was extracted from individual mosquitoes. Extraction was performed using the Magnetic Bead Micro Tissue Genomic DNA Extraction Kit II (BioTeke, Wuxi, China) following the manufacturer’s instructions. The extracted DNA was subsequently used for *Wolbachia* infection detection and amplification of the mitochondrial ND5 gene fragment.

### 2.3 Amplification and sequencing of *Wolbachia wsp* and ND5

*Wolbachia* infection was detected by Polymerase Chain Reaction (PCR) using *Wolbachia* surface protein (*wsp*) encoding gene-based typing primers (Dobson et al., 1999; Ma et al., 2025). The primers used for *w*AlbA typing were wsp136F: 5′-TGAAATTTTACCTCTTTTC-3′ and wsp691R: 5′-AAAAATTAAACGCTACTCCA-3′, and the primers used for *w*AlbB typing were wsp183F: 5′-AAGGAACCGAAGTTCATG-3′ and wsp691R: 5′-AAAAATTAAACGCTACTCCA-3′. Coinfection with *w*AlbA and *w*AlbB (coinfection A&B) was defined as the simultaneous presence of both strains in the same sample. The mitochondrial ND5 gene fragment was amplified using primers ND5-F: 5′-TCCTTAGAATAAAATCCCGC-3′ and ND5-R: 5′-GTTTCTGCTTTAGTTCATTCTTC-3′.

PCR were performed in a 25 μL system, comprising 12.5 μL of Premix Taq (TaKaRa, Beijing, China), 9.5 μL of nuclease-free water, 1 μL each of forward and reverse primers, and 1 μL of DNA template. The amplification programs for *wsp* were as follows: initial denaturation at 94 °C for 5 min; 35 cycles of 94 °C for 1 min, annealing for 1 min, and 72 °C for 1 min; followed by a final extension at 72 °C for 10 min. The annealing temperatures for *w*AlbA and *w*AlbB were 45 °C and 48 °C, respectively. The amplification programs for ND5 were as follows: initial denaturation at 94 °C for 5 min; 35 cycles of 94 °C for 30 s, 56 °C for 45 s, and 72 °C for 1 min; followed by a final extension at 72 °C for 5 min. The amplification products were analyzed via 1% agarose gel electrophoresis with GelStain (TransGen, Beijing, China), purified via the Mini BEST Kit (TaKaRa, Beijing, China), and then sequenced at Shenzhen BGI Co., LTD (Shenzhen, China). The raw FASTA sequences of *w*AlbA, *w*AlbB and ND5 were submitted to the GenBank database.

### 2.4 Phylogenetic analysis and haplotype network construction

Multiple sequence alignment was performed using MUSCLE algorithm in MEGA 12 (Kumar et al., 2024). Phylogenetic trees were constructed using the maximum likelihood method with the Tamura-Nei model, and bootstrap support was assessed with 1,000 replicates, with complete deletion for missing/gap sites (Kumar et al., 2024). For the *wsp* phylogeny, 9 reference sequences were used, including *w*AlbA AF020058, *w*AlbA JX129187.1, *w*AlbA KC004024.1, *w*AlbB MF989987.1, *w*Pip MK695176.1, *w*AegB MN307069.1, *w*AlbB AF020059, *w*AlbB MG765533, and *w*AlbB KX573030, with *Rickettsia japonica* as the outgroup; the *wsp* sequences obtained in this study were assigned to *w*AlbA or *w*AlbB based on clustering with the reference sequences. Haplotype networks were constructed based on all ND5 and *wsp* sequences aligned using the MUSCLE algorithm and grouped by city or *Wolbachia* infection status, using the TCS Network method in PopART (Leigh et al., 2015).

### 2.5 Evolutionary association analysis and test for selective sweeps

First, the evolutionary associations between host ND5 haplotypes and *Wolbachia wsp* haplotypes were analyzed and visualized using the phytools package in R (Revell, 2024). Specifically, based on sample-level matching between ND5 haplotypes and *wsp* haplotypes, an association matrix was constructed using cophylo, and the association plot was generated using the plot.cophylo method, with the link.col. parameter distinguishing *w*AlbA (red) from *w*AlbB (grey), and the link.lwd parameter reflecting the number of matched samples for each haplotype pair. Second, to evaluate the potential effects of different *Wolbachia* infection types on ND5 genetic diversity, an ND5 haplotype × *Wolbachia* infection type contingency table was constructed based on pairwise matching of these two parameters for each sample. Pearson’s *χ*^2^ test, Monte Carlo test, and Cramer’s V test were used to test for a statistical correlation between the two variables. Subsequently, DnaSP 6 was used to compare ND5 genetic diversity among different *Wolbachia* infection types (Rozas et al., 2017). Tajima’s D (TD) and Fu’s Fs (Fs) statistics were calculated, and coalescent simulations were performed to test whether ND5 sequences deviated from neutral evolution (Rozas et al., 2017). To reduce the impact of sample size differences among cities on genetic diversity analyses and neutrality tests, the samples from the 16 cities were down-sampled (accommodating the city with the smallest sample size) and re-analyzed. Finally, AMOVA (analysis of molecular variance) was performed using Arlequin v3.1.0.2 to quantify the composition and proportion of genetic variation within and among populations, with samples from different sampling cities treated as distinct populations within each *Wolbachia* infection-status group (Excoffier et al., 2007).

### 2.6 Data processing and statistical analysis

City-level monthly average climatic data (temperature and precipitation) for the sample collection period, which also corresponded to the mosquito activity season (May to October 2024), were obtained from the official website of the Shandong Provincial Bureau of Statistics^1^ and used in this study (Supplementary Tables S2, S3). GraphPad Prism 8.0, QGIS 3.44.5, MEGA 12, PopART and phytools were used for data processing and visualization. SPSS 26 was used for statistical analysis. Spearman test, linear regression analysis, Pearson’s *χ*^2^ test, Monte Carlo test, and Cramer’s *V* test were used to analyze correlations; Fisher’s exact test was used to compare rates. *p* < 0.05 was considered statistically significant.

## 3 Results

### 3.1 Prevalence of *Wolbachia* in wild-caught *Ae. albopictus*

A total of 388 *Ae. albopictus* individuals were collected from 16 cities in Shandong Province and screened for *Wolbachia* infection using the rapidly evolving *wsp*-encoding gene. The overall *Wolbachia* infection rate was 92.8%, with 5.2% of individuals exhibited “*w*AlbA only” infection (detected in 7 cities), 6.2% exhibited “*w*AlbB only” infection (detected in 9 cities), and 81.4% exhibited coinfection A&B (detected in all 16 cities; Table 1). At the city level, the overall *Wolbachia* infection rate ranged from 61.5 to 100%. At the climatic region level, the infection rate showed a trend of eastern Shandong (98.7%) > central Shandong (97.1%) > northwestern Shandong (93.9%) > southwestern Shandong (92.7%) > south-central Shandong (77.6%; Table 1). The south-central climatic region showed the lowest overall infection rate, the lowest coinfection A&B rate, and the highest “*w*AlbA only” infection rate, with all differences being statistically significant (Table 1).

**Table 1:** *Wolbachia* infection rate and infection types in *Ae. albopictus* captured in Shandong Province.

Region	City	Sample size	Infected		*w*AlbA only		*w*AlbB only		Coinfection A&B	
			Positive	Rate (%)	Positive	Rate (%)	Positive	Rate (%)	Positive	Rate (%)
Eastern	YT	28	28	100^a^	0	0^a^	0	0^a^	28	100^a^
	QD	28	28	100^a^	0	0^a^	1	3.6^a^	27	96.4^ac^
	WH	20	19	95^ab^	1	5^a^	0	0^a^	18	90^abc^
	Total	76	75	98.7^A^	1	1.3^B^	1	1.3^A^	73	96.1^A^
Central	ZB	28	28	100^a^	0	0^a^	0	0^a^	28	100^a^
	JNA	26	26	100^ab^	0	0^a^	1	3.8^a^	25	96.2^abc^
	TA	24	23	95.8^ab^	1	4.2^a^	4	16.7^a^	18	75^abc^
	WF	27	25	92.6^ab^	1	3.7^a^	0	0^a^	24	88.9^abc^
	Total	105	102	97.1^A^	2	1.9^B^	5	4.8^A^	95	90.5^AB^
Northwestern	DY	27	27	100^a^	0	0^a^	0	0^a^	27	100^a^
	BZ	24	24	100^ab^	1	4.2^a^	3	12.5^a^	20	83.3^abc^
	DZ	25	23	92^ab^	2	8^a^	4	16^a^	17	68^abcd^
	LC	23	19	82.6^ab^	0	0^a^	4	17.4^a^	15	65.2^abcd^
	Total	99	93	93.9^A^	3	3.0^B^	11	11.1^A^	79	79.8^B^
Southwestern	JNI	29	29	100^a^	0	0^a^	0	0^a^	29	100^a^
	HZ	12	9	75^ab^	0	0^a^	2	16.7^a^	7	58.3^bcd^
	Total	41	38	92.7^AB^	0	0^B^	2	4.9^A^	36	87.8^AB^
South-Central	RZ	11	11	100^ab^	0	0^a^	0	0^a^	11	100^abc^
	LY	30	25	83.3^ab^	7	23.3^a^	1	3.3^a^	17	56.7^bd^
	ZZ	26	16	61.5^b^	7	26.9^a^	4	15.4^a^	5	19.2^d^
	Total	67	52	77.6^B^	14	20.9^A^	5	7.5^A^	33	49.3^C^
Total		388	360	92.8	20	5.2	24	6.2	316	81.4

Sample size refers to the total number of mosquitoes tested per city. Infected rate was calculated as (*w*AlbA only + *w*AlbB only + Coinfection A&B)/Number × 100%. Different lowercase letters (i.e., a, b, c, d) indicated significant differences in positive rates between different cities, and different uppercase letters (i.e., A, B, C) indicated significant differences in positive rates between different regions (Fisher’s exact test, *p* < 0.05). YT, Yantai; QD, Qingdao; WH, Weihai; ZB, Zibo; JNA, Jinan; TA, Taian; WF, Weifang; DY, Dongying; BZ, Binzhou; DZ, Dezhou; LC, Liaocheng; JNI, Jining; HZ, Heze; RZ, Rizhao; LY, Linyi; ZZ, Zaozhuang.

### 3.2 Geographical distribution and climatic responses of *Wolbachia*

The overall *Wolbachia* infection rate and infection types exhibited spatial heterogeneity across the 16 cities. Regarding the infection rate, eight cities (JNI, YT, DY, ZB, RZ, QD, JNA and BZ) showed an infection rate of 100%; Four cities (TA, WH, WF and DZ) had infection rates between 90 and 100%; Two cities (LY and LC) had infection rates between 80 and 90%; HZ exhibited an infection rate of 75%; and ZZ had the lowest infection rate at 61.5% (Figure 1A). The statistically significant differences in infection rates between pairwise cities were presented in Table 1. Regarding the infection types, five cities (BZ, TA, DZ, LY and ZZ) had “*w*AlbA only” infection, “*w*AlbB only” infection and coinfection A&B; Four cities (QD, JNA, LC and HZ) had “*w*AlbB only” infection and coinfection A&B, while two cities (WH and WF) had “*w*AlbA only” infection and coinfection A&B. Interestingly, all tested mosquitoes from the five cities with 100% overall positive rates (JNI, YT, DY, ZB and RZ) were found to be coinfection A&B (Figure 1A). Spearman correlation analysis showed that the overall *Wolbachia* infection rate across the 16 cities was significantly positively correlated with the coinfection A&B rate (rho = 0.893, *p* < 0.001), while significantly negatively correlated with the “*w*AlbA only” infection rate (rho = −0.557, *p* = 0.025) and the “*w*AlbB only” infection rate (rho = −0.581, *p* = 0.018). To examine the contribution of temperature and precipitation to infection rates, linear regression and spearman correlation analyses were performed. The results showed that the overall *Wolbachia* infection rate and the coinfection A&B rate were significantly negatively correlated with temperature (rho = −0.476, *p* = 0.031; rho = −0.440, *p* = 0.044), while the “*w*AlbA only” infection rate was significantly positively correlated with precipitation (rho = 0.693, *p* = 0.001; Figure 1B).

**Figure 1:**
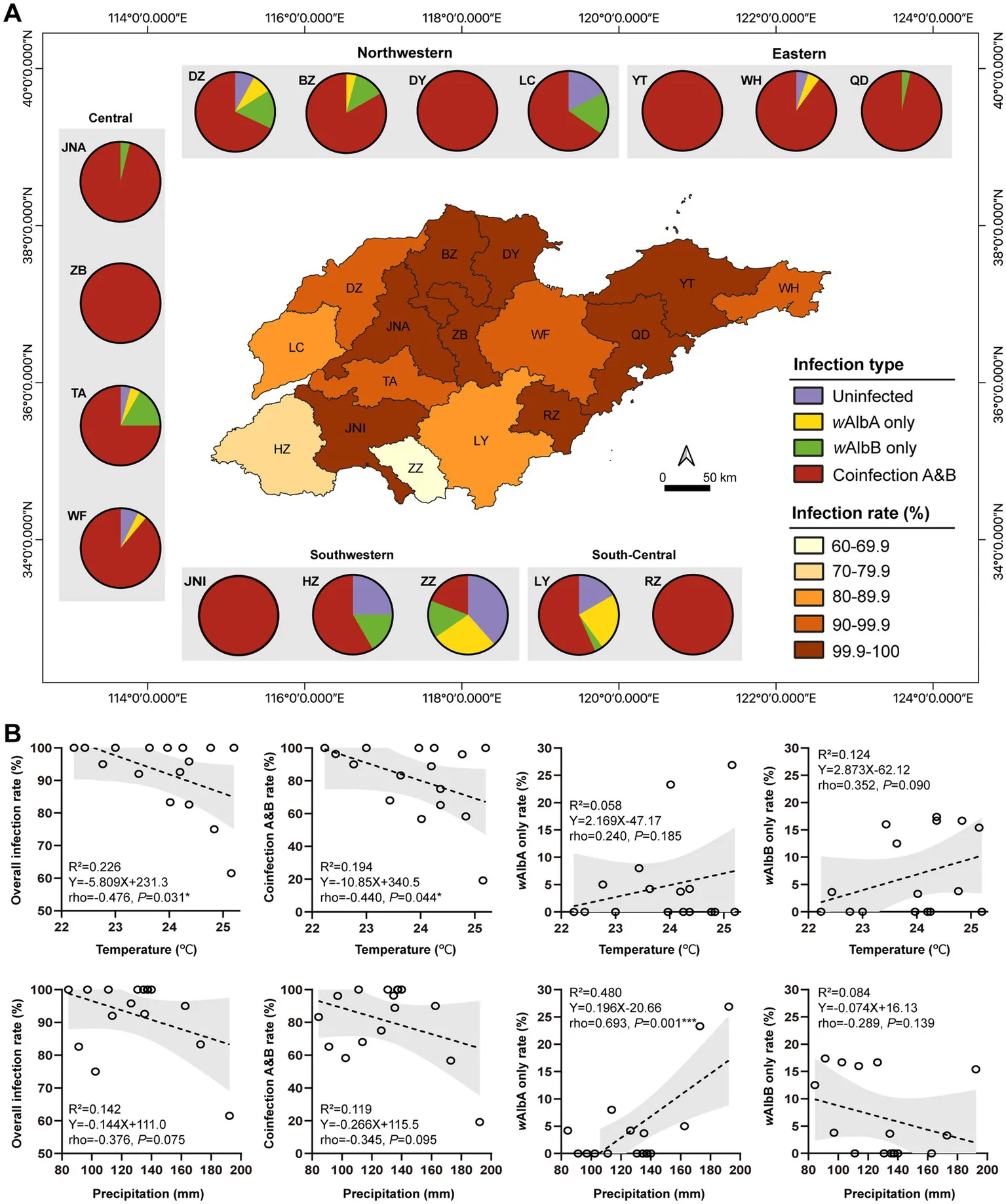
Geographic distribution (**A**) and climatic responses (**B**) of *Wolbachia* infection rates and infection types in 16 *Ae. albopictus* populations captured in Shandong Province. The city-level average temperature and precipitation during the sampling period (May–October) were used for correlation and regression analyses with the *Wolbachia* infection rates. YT, Yantai; QD, Qingdao; WH, Weihai; ZB, Zibo; JNA, Jinan; TA, Taian; WF, Weifang; DY, Dongying; BZ, Binzhou; DZ, Dezhou; LC, Liaocheng; JNI, Jining; HZ, Heze; RZ, Rizhao; LY, Linyi; ZZ, Zaozhuang.

### 3.3 Haplotype network and phylogenetic analysis of *Wolbachia* and ND5

All *wsp* sequences obtained in this study could be broadly classified into *w*AlbA (GenBank accession No.: PZ751068-PZ751392 and PZ752121-PZ752131) and *w*AlbB (GenBank accession No.: PZ751393-PZ751723 and PZ752112-PZ752120). *w*AlbA included one haplotype with an Hd value of 0 and a *π* value of 0. *w*AlbB included four haplotypes with a Hd value of 0.024 and a *π* value of 0.00007 (Figure 2A). Phylogenetic analysis revealed that all the sequences clustered into two major branches: *w*AlbA and *w*AlbB. The *w*AlbA sequences obtained in this study were closely related to the *w*AlbA AF020058, *w*AlbA JX129187.1 and *w*AlbA KC004024.1 subgroup. The *w*AlbB sequences obtained in this study were closely related to the *w*AlbB MF989987.1, *w*Pip MK695176.1, *w*AegB MN307069.1, *w*AlbB AF020059, *w*AlbB MG765533 and *w*AlbB KX573030 subgroups (Figure 2B).

**Figure 2:**
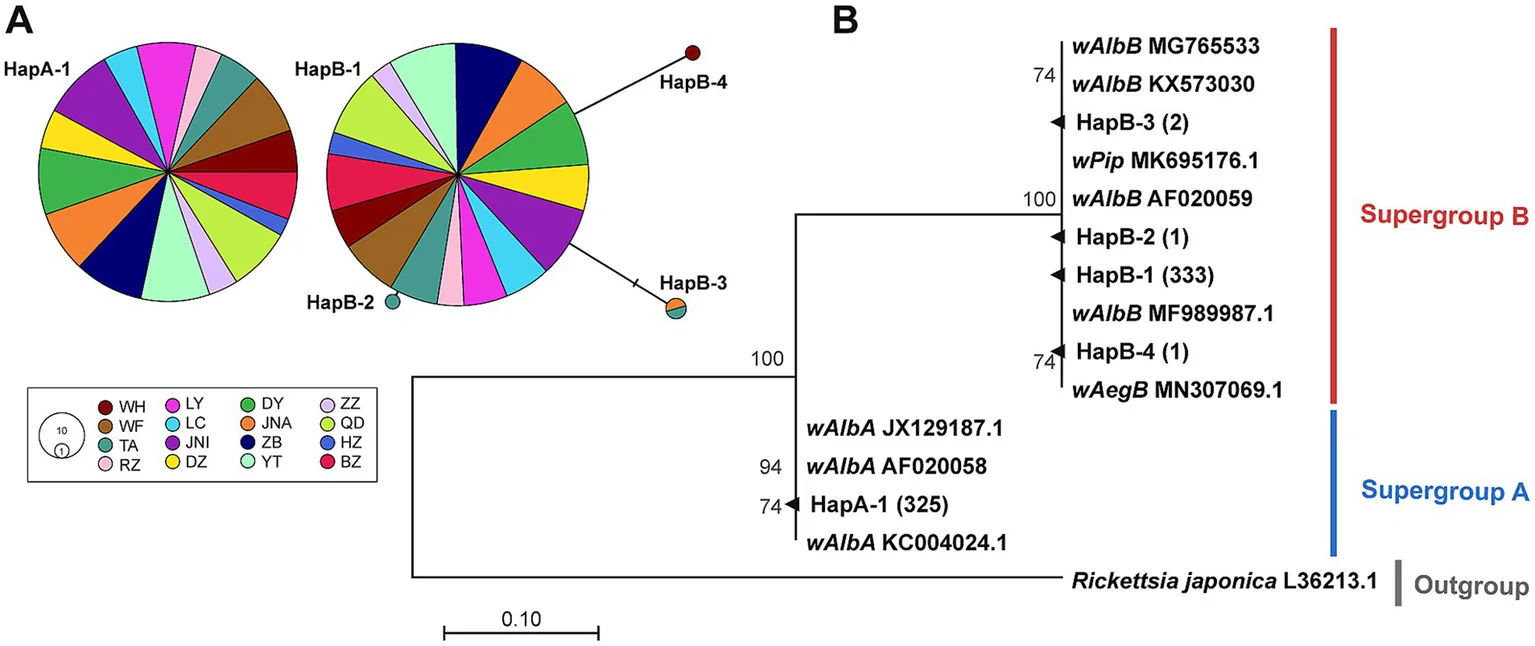
Haplotype network **(A)** and phylogenetic analysis **(B)** based on *Wolbachia wsp* sequences. In **A**, the circles represented different haplotypes, the circle size represented the number of haplotypes, and the short lines on the branches represented the number of mutation steps. In **B**, the numbers in parentheses indicated the number of sequences assigned to corresponding haplotype.

After alignment and removal of gap-containing sites, a 387 bp sequence was ultimately obtained for ND5. Among the 388 ND5 sequences (GenBank accession No.: PZ751724-PZ752111), 66 polymorphic sites and 36 parsimony-informative sites were identified, defining 21 haplotypes. The overall haplotype diversity (Hd) was 0.592, nucleotide diversity (*π*) was 0.00331, and the average number of nucleotide differences (*K*) was 1.282. Haplotype network analysis indicated that Hap1 and Hap2 were the predominant haplotypes. Hap1 included 181 sequences and was distributed across all 16 cities, while Hap2 included 169 sequences and was found in 13 cities. The remaining haplotypes were low-frequency or private, with Hap16-Hap18 separated from other haplotypes by multiple mutational steps (Figure 3A). Phylogenetic analysis showed that Hap1-Hap15 and Hap18-Hap21 formed one clade, Hap17-Hap18 formed another, and Hap16 formed a separate lineage (Figure 3B). Among the predominant haplotypes, the *Wolbachia* infection rates in Hap1 and Hap2 were 90.6 and 95.9%, respectively. Among the low-frequency haplotypes, Hap9, Hap10, Hap14, and Hap18 had an *Wolbachia* infection rate of 0%, whereas all other low-frequency haplotypes showed 100% (Figure 3B). As most low-frequency haplotypes were represented by only a single individual, no clear correlation was observed between the phylogeny of ND5 haplotypes and *Wolbachia* infection rates.

**Figure 3:**
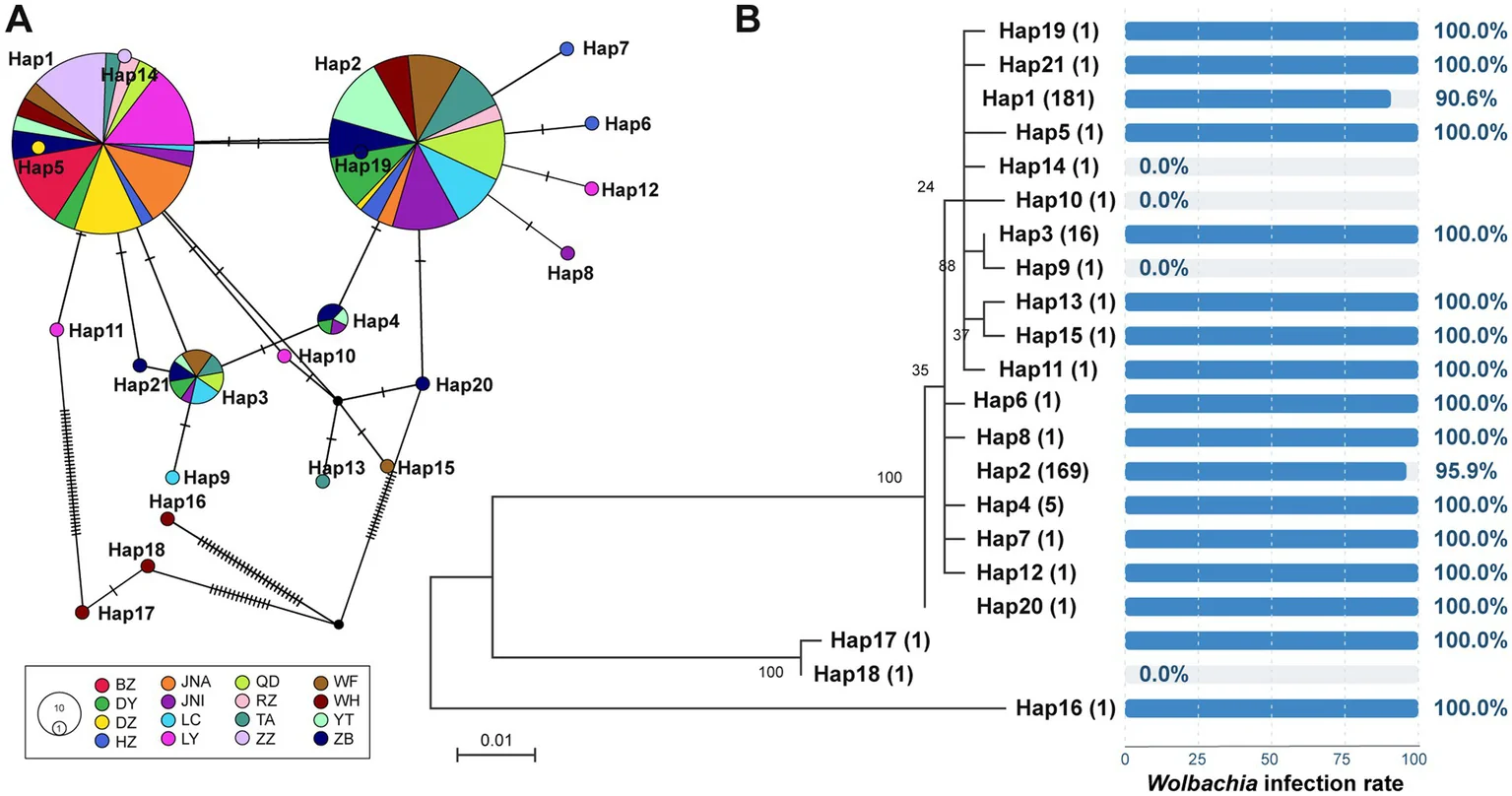
Haplotype network **(A)** and phylogenetic analysis **(B)** based on ND5 sequences. In **A**, the circles represented different haplotypes, the circle size represented the number of haplotypes, and the short lines on the branches represented the number of mutation steps. In **B**, the numbers in parentheses indicated the number of sequences assigned to corresponding haplotype. The *Wolbachia* infection rate was indicated following the corresponding haplotype.

### 3.4 Association between *Wolbachia* and ND5 haplotypes

To visualized the association between ND5 haplotypes and *Wolbachia* haplotypes at the sample level, an ND5-*Wolbachia* association plot was constructed based on individual-level matching results (Figure 4). The results showed relatively concentrated connections between the predominant ND5 haplotypes Hap1 and Hap2 and the predominant *Wolbachia* haplotypes HapA-1 and HapB-1, along with a few connections among other haplotypes, indicating that *Wolbachia* infection was not restricted to a single ND5 genetic background, but was scattered across and mainly enriched in the predominant ND5 haplotypes.

**Figure 4:**
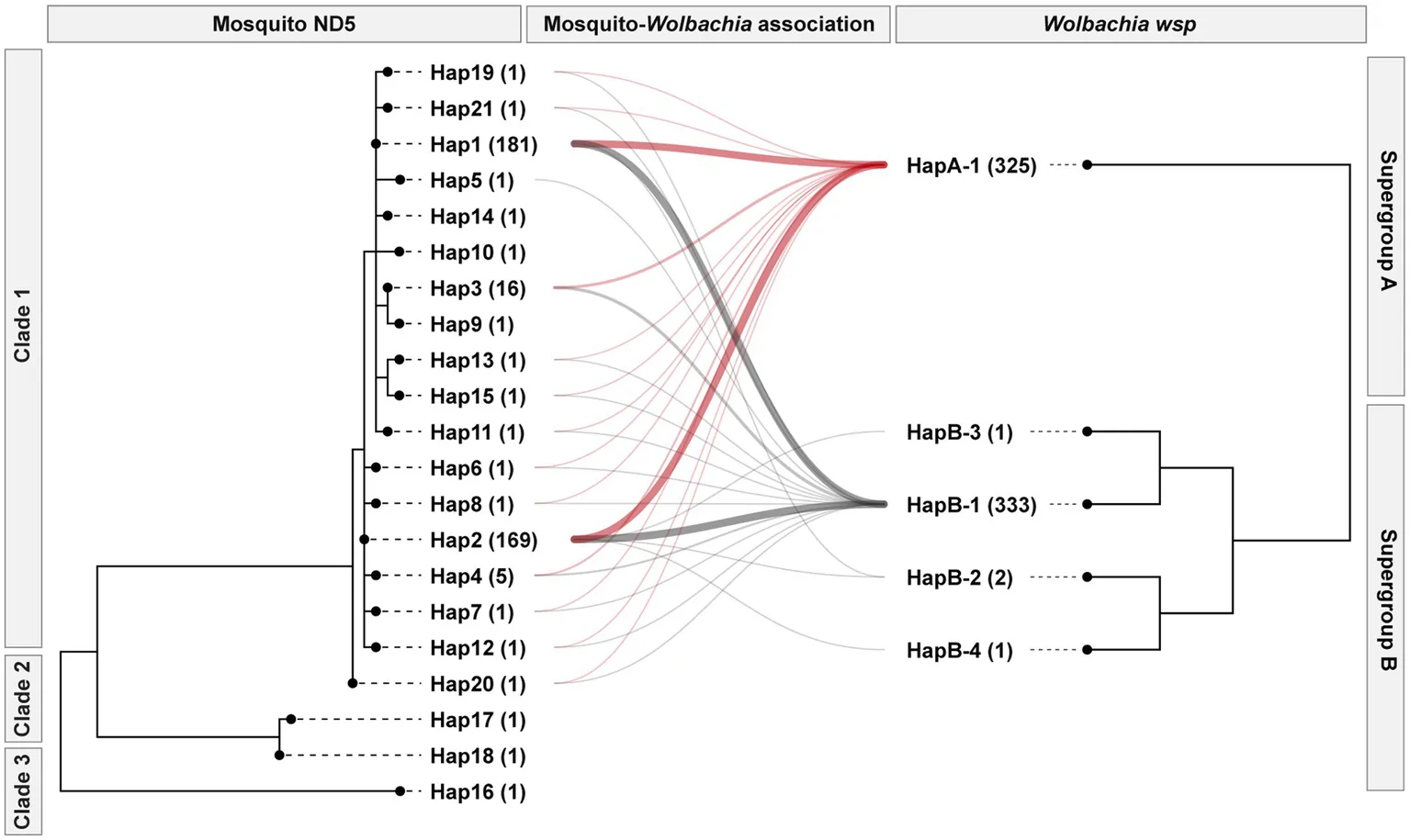
Tanglegram-style association between ND5 haplotypes and *Wolbachia wsp* haplotypes. The left panel showed the ND5 haplotypes, the right panel showed *Wolbachia* haplotypes, and the middle curves indicated individual-level matching results. Red lines indicated *w*AlbA associations and grey lines indicated *w*AlbB associations; Line thickness was proportional to the number of matched samples. Numbers after haplotypes indicated sample counts. ND5 haplotypes without a connecting line lacked a genotypable *Wolbachia wsp* sequence for association analysis. Among these, Hap9, Hap10, Hap14, and Hap18 corresponded to individuals confirmed *Wolbachia*-uninfected by PCR, whereas Hap16 and Hap17 corresponded to PCR-positive individuals for which a genotypable *wsp* sequence was not obtained.

### 3.5 Effects of different *Wolbachia* infection types on ND5 genetic diversity

Combined analysis of *Wolbachia* infection types and ND5 haplotypes revealed that the predominant ND5 haplotypes, Hap1 and Hap2, each contained all four infection types: coinfection A&B, “*w*AlbA only” infection, “*w*AlbB only” infection, and uninfected individuals. Hap3 included two infection types: coinfection A&B and “*w*AlbB only” infection, whereas Hap4-Hap21 each contained only one infection type (Figure 5). An original contingency table was constructed based on 21 ND5 haplotypes and 4 *Wolbachia* infection types (Table 2), and the chi-square test showed that the two variables were significantly correlated (*χ*^2^ = 113.806, d.f. = 60, *p* = 3.43 × 10^−5^). Given the presence of multiple low-frequency haplotypes and some low expected counts, a Monte Carlo exact test was further performed, which also reached statistical significance (*p* = 0.0241). Cramer’s *V* was 0.313, indicating a moderate correlation between the two variables.

**Figure 5:**
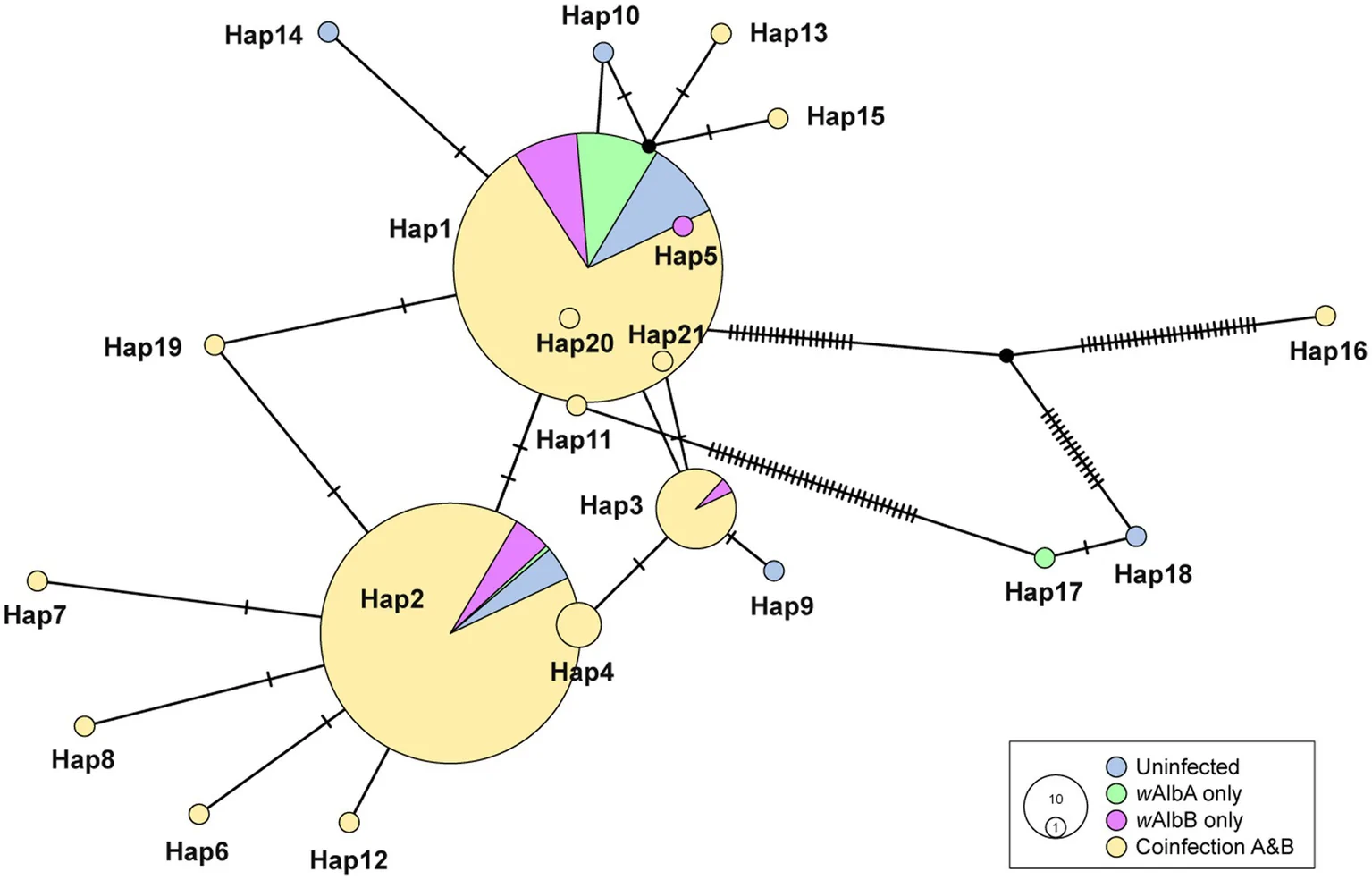
Haplotype network of ND5 haplotypes based on *Wolbachia* infection types. The circles represented different haplotypes, the circle size represented the number of haplotypes, the short lines on the branches represented the number of mutation steps, and different colors represented different *Wolbachia* infection types.

**Table 2:** ND5 haplotype × *Wolbachia* infection type in *Ae. albopictus.*

ND5 haplotype × *Wolbachia* infection type	Uninfected	*w*AlbA only	*w*AlbB only	Coinfection A&B	Total
Hap1	17	18	14	132	181
Hap2	7	1	8	153	169
Hap3	0	0	1	15	16
Hap4	0	0	0	5	5
Hap5-Hap21	4	1	1	11	17
Total	28	20	24	316	388

To further evaluated the potential impact of *Wolbachia* infection on ND5 variation, samples were grouped according to their *Wolbachia* infection status into the uninfected group, infected group, “*w*AlbA only” infection group, “*w*AlbB only” infection group, and coinfection A&B group. ND5 genetic diversity estimate and neutrality test were then performed for each group. Compared with the uninfected group, the infected group, “*w*AlbB only” infection group and coinfection A&B group showed similar levels of ND5 haplotype diversity but markedly reduced nucleotide diversity, whereas the “*w*AlbA only” infection group exhibited substantially lower ND5 haplotype diversity but higher nucleotide diversity (Table 3). After balanced down-sampling across cities, similar trends were observed (Supplementary Table S4).

**Table 3:** ND5 genetic diversity among different *Wolbachia* infection type groups.

Group	*N*	H	Hd	*π*	*K*	S	Fs	Fs *p* value	TD	TD *p* value
Uninfected	28	6	0.585	0.00826	3.206	39	1.855	0.143	−2.56548	<0.001
Infected	360	17	0.589	0.00294	1.140	66	−8.291	0.000	−2.65021	<0.001
*w*AlbA only	20	3	0.195	0.00923	3.600	36	5.788	0.016	−2.55653	<0.001
*w*AlbB only	24	4	0.569	0.00183	0.714	4	−0.597	0.210	−0.91248	>0.10
Coinfection A&B	316	15	0.590	0.00258	1.002	55	−7.443	0.000	−2.58911	<0.001

*N*, sample size; H, number of haplotypes; Hd, haplotype diversity; *π*, nucleotide diversity; *K*, average number of nucleotide differences; S, number of segregating sites; TD, Tajima’s D statistic; Fs, Fu’s Fs statistic.

Neutrality tests showed that TD and Fs were negative in the infected group, the coinfection A&B group, and the “*w*AlbB only” infection group (Table 3). Specifically, the infected group showed more negative TD and Fs values than the uninfected group, and the coinfection A&B group exhibited more negative and more statistically significant TD and Fs values than the single-infection groups (Table 3). Additionally, the AMOVA showed that the ND5 genetic variation in the infected group, the coinfection A&B group and the “*w*AlbB only” infection group was mainly distributed within populations, whereas in the “*w*AlbA only” infection group and uninfected group, it was mainly distributed among populations (Table 4).

**Table 4:** AMOVA comparing ND5 genetic variation among different *Wolbachia* infection types.

Group	*N*	Source of variation	d.f.	Sum of squares	Variance component	Percentage of variation (%)
Uninfected	28	Among populations	7	45.974	1.75851 Va	64.47
		Within populations	20	19.383	0.96917 Vb	35.53
		Total	27	65.357	2.72768	/
Infected	360	Among populations	15	100.612	0.26017 Va	22.81
		Within populations	344	302.932	0.88062 Vb	77.19
		Total	359	403.544	1.14079	/
*w*AlbA only	20	Among populations	6	40.950	2.47174 Va	76.27
		Within populations	13	10.000	0.76923 Vb	23.73
		Total	19	50.950	3.24097	/
*w*AlbB only	24	Among populations	8	12.625	0.35497 Va	34.94
		Within populations	15	9.917	0.66111 Vb	65.06
		Total	23	22.542	1.01608	/
Coinfection A&B	316	Among populations	15	73.822	0.21023 Va	20.65
		Within populations	300	242.425	0.80808 Vb	79.35
		Total	315	316.247	1.01832	/
Total	388	Among populations	15	117.699	0.28497 Va	22.91
		Within populations	372	356.696	0.95886 Vb	77.09
		Total	387	474.394	1.24383	/

*N*, sample size; d.f., degrees of freedom; Va, among-population variance component; Vb, within-population variance component; fixation index refers to FST. AMOVA was performed by treating the 16 sampling cities/sites as populations within each infection-status group.

## 4 Discussion

In our study, *Wolbachia* infection was prevalent in female *Ae. albopictus* from Shandong Province, with coinfection A&B as the dominant type, while single infections were relatively rare. Globally, *Wolbachia* infection rates in female *Ae. albopictus* were typically above 90%, and in some regions approach 100%, for example, Greece (97%) (Michail et al., 2024), Hawaii (98.5%) (Seok et al., 2026), Malaysia (98.6%) (Ahmad et al., 2017), and Iran (100%) (Ghavami et al., 2025). However, lower infection rates have also been reported in some areas. For instance, Karimian et al. found that *Wolbachia* infection rates in *Ae. albopictus* collected from three northern provinces of Iran ranged from only 0 to 17% (Karimian et al., 2026). In China, the *Wolbachia* infection rates in female *Ae. albopictus* ranged from 85.7 to 100% in different cities and districts of Jiangsu Province, 79.3–100% in Anhui, 85.7–93.3% in Jiangxi, 86.7–100% in Hubei, 92.6–100% in Chongqing, 95.5–100% in Sichuan, and 84–91.08% in Shanghai (Ma et al., 2025; Liu et al., 2026). Compared with the above provinces and municipalities, the *Wolbachia* infection rate in Shandong showed greater variation among cities (61.5–100%), and this variation was also evident across the five climatic regions (77.6–98.7%). In summary, the prominent feature of *Wolbachia* infection in Shandong was not a uniformly high prevalence across the entire province, but rather a pattern of notable inter-city differences and regional heterogeneity against a backdrop of overall high rates.

Temperature and precipitation have been recognized as important abiotic regulators of *Wolbachia* infection in hosts (Alonso et al., 2022). Carlassara et al. found that constant exposure to 18 °C during *Ae. albopictus* developmental stages induced a decrease in the density of the native *Wolbachia* strains, *w*AlbA and *w*AlbB, compared to those reared at 28 and 32 °C (Carlassara et al., 2024). Studies on field populations of *Ae. albopictus* in Greece showed a significant positive correlation between temperature and the prevalence of single infections (*p* = 6.498 × 10^−7^) (Michail et al., 2024). In field populations of *Ae. albopictus* in Hawaii, the prevalence of *w*AlbA was positively correlated with temperature and precipitation, while the prevalence of *w*AlbB was influenced by island location and precipitation (Seok et al., 2026). In our study, the “*w*AlbA only” infection rate was significantly positively correlated with precipitation, and both “*w*AlbA only” and “*w*AlbB only” infection rates showed weak positive correlations with temperature, which was largely consistent with the findings of the above-mentioned studies. We also found that the overall *Wolbachia* infection rate and the coinfection A&B rate were significantly negatively correlated with temperature in Shandong Province. Furthermore, among the five cities where both “*w*AlbA only” and “*w*AlbB only” infections were present, the two cities with a higher “*w*AlbA only” than “*w*AlbB only” infection rate (LY and ZZ) were both located in the South-Central Shandong climatic region, whereas the three cities with a higher “*w*AlbB only” than “*w*AlbA only” infection rate (BZ, DZ, and TA) were all distributed in the Central and Northwestern Shandong climatic regions. Whether this phenomenon was random or driven by other unknown factors remained to be further investigated (Alonso et al., 2022).

*Wolbachia*, as an intracellular endosymbiont of mosquitoes, not only regulates host reproduction through cytoplasmic incompatibility, but also affects mosquito fitness and vector competence. Therefore, differential risks posed by varying *Wolbachia* infection rates and infection types in different cities warrant attention. Laboratory studies showed that, under standard rearing conditions, naturally *Wolbachia*-infected female *Ae. albopictus* exhibited higher fecundity and longer longevity than uninfected individuals, and that coinfected individuals exhibited higher fecundity and longer longevity than either uninfected or singly infected individuals (Dobson et al., 2001; Dobson et al., 2004). Therefore, female *Ae. albopictus* in cities with high infection rates, especially those with high coinfection A&B rates (e.g., JNI, YT, ZB, DY, and RZ), may benefit in terms of fitness. This fitness advantage, together with multiple ecological factors—including breeding site availability, predation, insecticide use, and interspecific competition—may collectively shape field population density. Furthermore, *Wolbachia* infection may potentially influence the vector competence of *Ae. albopictus* for arboviruses. Although most studies showed that natural *Wolbachia* infection had limited inhibitory effects on the transmission of DENV, ZIKV, and CHIKV by *Ae. albopictus*—likely because the *Wolbachia* densities in somatic tissues (e.g., midgut, fat body, and salivary glands) were below the threshold required to induce a potent antiviral response—some studies demonstrated that naturally occurring *Wolbachia* in *Ae. albopictus* could partially inhibit the dissemination of DENV-2 and CHIKV to the salivary glands (Mousson et al., 2012; Ahmad et al., 2017). Tsai et al. found that after oral infection with DENV-1, *Wolbachia*-infected *Ae. albopictus* carried lower viral titers than *Wolbachia*-free mosquitoes (Tsai et al., 2017). Khan et al. reported that after oral infection with DENV-2, the viral titers were negatively correlated with the density of naturally infected *Wolbachia* (*w*AlbA and *w*AlbB) in *Ae. albopictus*, suggesting that naturally infected *Wolbachia* had a potential inhibitory effect on viral replication (Khan et al., 2025). In this study, the natural *Wolbachia* infection rate in *Ae. albopictus* from southern Shandong (especially in ZZ and HZ) was relatively low. Whether this has a potential impact on local vector competence remains to be further clarified, as direct phenotypic data on viral loads were not obtained in this study.

*Wolbachia* was reported to influence the genetic structure of host populations through cytoplasmic hitchhiking or indirect selective sweeps during co-inheritance with the host (Kambhampati et al., 1992; Jiggins, 2003). Specifically, once a population became infected with *Wolbachia*, due to the matrilineal inheritance of the host’s mtDNA and *Wolbachia*, as well as the fitness advantage of the *Wolbachia*-infected host, the mtDNA haplotypes related to *Wolbachia* were more likely to spread within the population, gradually replacing other haplotypes and becoming the predominant haplotypes, thereby leading to a decrease in the haplotype diversity of the population (Dean et al., 2003; Shoemaker et al., 2004). Our findings corroborated this view. In our study, the genetic diversity of *Wolbachia* was low, with only one haplotype detected for *w*AlbA (Hd = 0, *π* = 0) and four haplotypes for *w*AlbB (Hd = 0.024, *π* = 0.00007). The mitochondrial ND5 gene also exhibited relatively low genetic diversity, with 21 haplotypes identified in total (Hd = 0.592, *π* = 0.00331), of which 90.206% (350/388) of individuals belonged to the predominant haplotypes Hap1 and Hap2. Phylogenetic analysis of ND5 revealed that all haplotypes clustered into three clades, with the predominant haplotypes Hap1 and Hap2 located in the largest clade, both showing high *Wolbachia* infection rates. The reduction of mtDNA diversity induced by *Wolbachia* infection was also observed in other mitochondrial genes and mosquito species. Ma et al. reported that in 22 *Ae. albopictus* populations from the Yangtze River Basin of China, the overall *Wolbachia* infection rate was high (94.2%), and the COI gene haplotype diversity was low (Hd = 0.261, *π* = 0.00058), with 85.876% (456/531) of individuals belonging to the prodominant haplotype Hap1 (Ma et al., 2025). The Hd, *π*, and *K* values of the COI gene in the infected, singly infected, and doubly infected groups were all reduced compared with the uninfected group, with the doubly infected group showing lower values than the singly infected group (Ma et al., 2025). Similarly, Rasgon et al. found that in *Culex pipiens* complex populations, 99% of individuals in *Wolbachia*-infected populations carried a single ND4 haplotype, whereas uninfected populations retained a rich ancestral diversity (Rasgon et al., 2006). Given the potential for *Wolbachia*-mediated selective sweeps to obscure true evolutionary relationships, we recommend combining mitochondrial genes—whether ND5 or COI—with at least one independent nuclear genetic marker when studying closely related species complexes or populations with high *Wolbachia* infection prevalence, particularly for validating species boundaries.

Additionally, we found that different *Wolbachia* infection types might have differential effects on ND5 variation. We divided 16 natural populations of *Ae. albopictus* into four groups according to *Wolbachia* infection status (uninfected, infected, “*w*AlbA only” infected, “*w*AlbB only” infected, and coinfection A&B) and then analyzed mitochondrial ND5 diversity separately for each group. Compared with the uninfected group, the infected, “*w*AlbB only,” and coinfection A&B groups showed no marked changes in Hd, but their *π* and *K* values were notably lower, indicating that although these groups contained multiple ND5 haplotypes, sequence divergence among them was limited. Such a pattern is often associated with recent population expansion or the accumulation of low-frequency mutations over a short period (Grant and Bowen, 1998). This interpretation was further supported by the neutrality tests, which showed significantly negative TD and Fs values for both the infected and coinfection A&B groups (Fu, 1997). In contrast, the “*w*AlbA only” group had a relatively small sample size, with low Hd but high *π*, implying that while this group contained a limited number of haplotypes, these haplotypes may have been considerably divergent from one another. This was also supported by the AMOVA results, which showed a high proportion of among-population variation (76.27%) in this group, potentially reflecting the influence of local geographic population structure or a few highly divergent matrilineal lineages. However, given the small sample size, this interpretation should be treated with caution and requires validation with larger sample sizes in future studies. In addition, ND5 variation in the “*w*AlbB only” and coinfection A&B groups was mainly within populations, indicating shared predominant haplotypes across cities and some connectivity among populations, which might be associated with the strong adaptability and dispersal potential of *Ae. albopictus* as one of the most invasive mosquito species worldwide.

This study has three limitations that we document here to guide future research toward greater comprehensiveness and robustness. Firstly, the molecular data available for this study are limited. Ideally, integrating a broader range of mitochondrial markers (e.g., COI) along with nuclear genes as references would greatly strengthen the evidence for *Wolbachia*-associated selective sweeps and better confirm cytoplasmic hitchhiking effects. Second, the limited sample size constrains the strength of our inferences. The relatively small number of samples per city, as well as the limited sample sizes of the uninfected, “wAlbA only,” and “wAlbB only” groups, may affect the stability of genetic diversity estimates and AMOVA results. Third, the accuracy of the correlation analyses between *Wolbachia* infection and temperature and precipitation could be improved by employing meteorological data from the weather stations closest to the sampling locations, rather than relying on city-level monthly averages.

## 5 Conclusion

This study characterized the *Wolbachia* infection landscape and its effects on ND5 diversity in *Ae. albopictus* across Shandong Province. Infection rate was high with coinfection A&B predominant, yet varied notably among cities and climatic regions. Both *Wolbachia* and ND5 showed low genetic diversity. The decreased ND5 haplotype or nucleotide diversity associated with different *Wolbachia* infection types, along with negative neutrality test values, supported the occurrence of *Wolbachia*-induced selective sweeps on the host ND5 gene. Therefore, we recommend combining ND5 with at least one independent nuclear marker for species boundary validation in *Wolbachia*-rich systems. In future studies, integrating a broader range of mitochondrial and nuclear markers will further strengthen the evidence for *Wolbachia*-induced selective sweeps. This study improves our understanding of the mechanisms shaping *Ae. albopictus* population genetics and provides a foundation for vector surveillance and symbiont ecological assessment in Shandong. The heterogeneous infection patterns across cities suggest that region-specific approaches may be necessary for vector control strategies.

## Data Availability

The datasets presented in this study can be found in online repositories. The names of the repository/repositories and accession number(s) can be found in the article/Supplementary material.

## Footnotes

1 http://tjj.shandong.gov.cn/tjnj/nj2025/zk/indexch.htm.
