# Supplementary material for: Wolbachia infection and its effects on mitochondrial ND5 gene variation in Aedes albopictus from Shandong Province
Source: Front Microbiol. 2026 Oct 6;17:1943745. doi: 10.3389/fmicb.2026.1943745 (PMC13680165; doi:10.3389/fmicb.2026.1943745)
Supplement: Supplementary file 1 [file Supplementary_file_1.docx]

Supplementary Material

# 1 Supplementary Table

**Supplementary Table 1.** *Aedes albopictus* collected in Shandong Province from May to October 2024.

| City | Number | Longitude | Latitude | Date |
| --- | --- | --- | --- | --- |
| YT | 28 | 120.33 | 37.63 | 2024.8.15 |
| QD | 28 | 119.9 | 36.7 | 2024.10.13 |
| WH | 20 | 122.473 | 37.153 | 2024.9.20 |
| ZB | 28 | 117.98 | 36.66 | 2024.9.6 |
| JNA | 26 | 117.554722 | 36.788333 | 2024.5.22 |
| TA | 24 | 117.13 | 36.192 | 2024.10.26 |
| WF | 27 | 119.218889 | 36.479722 | 2024.10.20 |
| DY | 27 | 118.57212 | 37.572354 | 2024.10.31 |
| BZ | 24 | 117 | 36.81 | 2024.9.15 |
| DZ | 25 | 116.64 | 36.93 | 2024.8.28 |
| LC | 23 | 116.26 | 36.57 | 2024.6.22 |
| JNI | 29 | 117.139604 | 34.821259 | 2024.10.20 |
| HZ | 12 | 115.75 | 35.033333 | 2024.10.26 |
| RZ | 11 | 119.203371 | 35.823084 | 2024.6.8 |
| LY | 30 | 118.4834 | 35.5024 | 2024.6.7 |
| ZZ | 26 | 117.1659 | 35.1141 | 2024.7.2 |

Note: YT, Yantai; QD, Qingdao; WH, Weihai; ZB, Zibo; JNA, Jinan; TA, Taian; WF, Weifang; DY, Dongying; BZ, Binzhou; DZ, Dezhou; LC, Liaocheng; JNI, Jining; HZ, Heze; RZ, Rizhao; LY, Linyi; ZZ, Zaozhuang.

**Supplementary Table 2.** City-level monthly average temperature (℃) corresponding to the sample collection period (May to October 2024).

| City | May | Jun | Jul | Aug | Sep | Oct |
| --- | --- | --- | --- | --- | --- | --- |
| JNA | 23.4 | 29.5 | 27.9 | 28.4 | 23.4 | 16 |
| QD | 17.4 | 22.6 | 25.2 | 28.1 | 24.6 | 16.7 |
| ZB | 22 | 28.6 | 27.9 | 28.2 | 22.8 | 14.3 |
| ZZ | 23 | 28.2 | 28.1 | 29.7 | 25.6 | 16.3 |
| DY | 22 | 27.5 | 28 | 29 | 23.4 | 15.6 |
| YT | 18.8 | 23.3 | 25.7 | 27.3 | 22.8 | 15.5 |
| WF | 21.6 | 27 | 27.9 | 29 | 23.9 | 15.8 |
| JNI | 23.6 | 28.9 | 28.1 | 29.6 | 24.7 | 16.3 |
| TA | 23.1 | 28.2 | 27.3 | 28.6 | 24.1 | 14.9 |
| WH | 19 | 23.9 | 26 | 27.9 | 23.5 | 16.3 |
| RZ | 18.8 | 23.6 | 26.2 | 28.5 | 24.2 | 16.7 |
| LY | 21.7 | 26.3 | 27 | 28.6 | 24.2 | 16.3 |
| DZ | 21.4 | 28.1 | 27.6 | 27.5 | 21.9 | 14.1 |
| LC | 22.2 | 28.4 | 27.7 | 28.5 | 23.5 | 15.9 |
| BZ | 21.5 | 27.4 | 27.7 | 28.1 | 22.6 | 14.5 |
| HZ | 23.2 | 28.8 | 27.7 | 29 | 23.9 | 16.4 |

Note: YT, Yantai; QD, Qingdao; WH, Weihai; ZB, Zibo; JNA, Jinan; TA, Taian; WF, Weifang; DY, Dongying; BZ, Binzhou; DZ, Dezhou; LC, Liaocheng; JNI, Jining; HZ, Heze; RZ, Rizhao; LY, Linyi; ZZ, Zaozhuang.

**Supplementary Table 3.** City-level monthly precipitation (mm) corresponding to the sample collection period (May to October 2024).

| City | May | Jun | Jul | Aug | Sep | Oct |
| --- | --- | --- | --- | --- | --- | --- |
| JNA | 44.7 | 9.1 | 206.4 | 240.9 | 47.5 | 34.3 |
| QD | 48.9 | 78.3 | 399.4 | 110.5 | 25.6 | 145.1 |
| ZB | 8.3 | 14.6 | 370.3 | 191.9 | 36.7 | 45.3 |
| ZZ | 21.8 | 10.4 | 774.6 | 191.9 | 114.5 | 40.8 |
| DY | 20.9 | 15.5 | 340.8 | 379.8 | 22.2 | 46.1 |
| YT | 55 | 62.8 | 215.1 | 227.3 | 72.8 | 151.6 |
| WF | 28.4 | 17.8 | 437.1 | 230.7 | 37.2 | 61.3 |
| JNI | 19.8 | 15.3 | 516.9 | 121.9 | 121.7 | 44.5 |
| TA | 13.5 | 34.8 | 447 | 184 | 25.6 | 53.4 |
| WH | 51.4 | 18.8 | 335.1 | 241.6 | 207.2 | 121.7 |
| RZ | 33.5 | 49.1 | 445.2 | 89.2 | 77.5 | 128.9 |
| LY | 49.6 | 99 | 686.6 | 109.9 | 31.8 | 60.8 |
| DZ | 31 | 14.9 | 215.6 | 326.1 | 60.6 | 34.2 |
| LC | 15.8 | 30.1 | 309.7 | 142.6 | 18.8 | 30.3 |
| BZ | 21.4 | 10.8 | 196.8 | 218.5 | 23.8 | 34.6 |
| HZ | 39.4 | 19.8 | 382.1 | 73.8 | 41.4 | 58 |

Note: YT, Yantai; QD, Qingdao; WH, Weihai; ZB, Zibo; JNA, Jinan; TA, Taian; WF, Weifang; DY, Dongying; BZ, Binzhou; DZ, Dezhou; LC, Liaocheng; JNI, Jining; HZ, Heze; RZ, Rizhao; LY, Linyi; ZZ, Zaozhuang.

**Supplementary Table 4.** ND5 genetic diversity among different *Wolbachia* infection type groups after down-sampling to accommodate the city with the smallest sample size.

| Group | N | H | Hd | π | K | S | TD |
| --- | --- | --- | --- | --- | --- | --- | --- |
| Uninfected | 13 (9-18) | 4 (2-6) | 0.621 (0.439-0.758) | 0.0122 (0.0012-0.0197) | 4.75 (0.47-7.67) | 36 (1-39) | -1.987 (-2.351-1.376) |
| Infected | 163 (158-167) | 10 (7-14) | 0.590 (0.561-0.617) | 0.0032 (0.0017-0.0045) | 1.25 (0.65-1.74) | 52 (6-65) | -2.597 (-2.642- -0.758) |
| *w*AlbA only | 8 (5-12) | 2 (1-3) | 0.250 (0-0.600) | 0.0163 (0-0.0308) | 6.36 (0-12.0) | 35 (0-36) | -1.875 (-2.253- -0.864) |
| *w*AlbB only | 12 (7-16) | 3 (2-4) | 0.591 (0.409-0.715) | 0.0018 (0.0011-0.0029) | 0.69 (0.41-1.13) | 2 (1-4) | -0.290 (-1.322-1.475) |
| Coinfection A&B | 143 (137-148) | 9 (7-12) | 0.592 (0.558-0.621) | 0.0034 (0.0016-0.0037) | 1.31 (0.64-1.44) | 51 (4-55) | -2.617 (-2.653- -0.250) |
| Total | 176 (176-176) | 12 (9-16) | 0.593 (0.564-0.621) | 0.0038 (0.0018-0.0053) | 1.47 (0.68-2.04) | 53 (8-65) | -2.532 (-2.629- -1.188) |

Notes: N, sample size; H, number of haplotypes; Hd, haplotype diversity; π, nucleotide diversity; K, average number of nucleotide differences; S, number of segregating sites; TD, Tajima’s D statistic. Down-sampling was performed at a fixed size of n = 11 per city to accommodate the sample size of RZ, resulting in a total of 176 samples analyzed. Values in the table are presented as medians with 2.5%–97.5% empirical percentile intervals based on 1000 down-sampling replicates.
